# Impact of early phase COVID-19 precautionary behaviors on seasonal influenza in Hong Kong: A time-series modeling approach

**DOI:** 10.3389/fpubh.2022.992697

**Published:** 2022-11-14

**Authors:** Chun-Pang Lin, Ilaria Dorigatti, Kwok-Leung Tsui, Min Xie, Man-Ho Ling, Hsiang-Yu Yuan

**Affiliations:** ^1^School of Data Science, City University of Hong Kong, Kowloon, Hong Kong SAR, China; ^2^Department of Statistics, School of Arts and Sciences, Rutgers University, New Brunswick, NJ, United States; ^3^MRC Centre for Global Infectious Disease Analysis, School of Public Health, Imperial College London, London, United Kingdom; ^4^Grado Department of Industrial and Systems Engineering, College of Engineering, Virginia Polytechnic Institute and State University, Blacksburg, VA, United States; ^5^Department of Mathematics and Information Technology, Faculty of Liberal Arts and Social Sciences, The Education University of Hong Kong, Tai Po, Hong Kong SAR, China; ^6^Department of Biomedical Sciences, Jockey Club College of Veterinary Medicine and Life Sciences, City University of Hong Kong, Kowloon, Hong Kong SAR, China

**Keywords:** COVID-19, influenza, face mask, social distancing, time-series analysis, infectious disease modeling

## Abstract

**Background:**

Before major non-pharmaceutical interventions were implemented, seasonal incidence of influenza in Hong Kong showed a rapid and unexpected reduction immediately following the early spread of COVID-19 in mainland China in January 2020. This decline was presumably associated with precautionary behavioral changes (e.g., wearing face masks and avoiding crowded places). Knowing their effectiveness on the transmissibility of seasonal influenza can inform future influenza prevention strategies.

**Methods:**

We estimated the effective reproduction number (*R*_*t*_) of seasonal influenza in 2019/20 winter using a time-series susceptible-infectious-recovered (TS-SIR) model with a Bayesian inference by integrated nested Laplace approximation (INLA). After taking account of changes in underreporting and herd immunity, the individual effects of the behavioral changes were quantified.

**Findings:**

The model-estimated mean *R*_*t*_ reduced from 1.29 (95%CI, 1.27–1.32) to 0.73 (95%CI, 0.73–0.74) after the COVID-19 community spread began. Wearing face masks protected 17.4% of people (95%CI, 16.3–18.3%) from infections, having about half of the effect as avoiding crowded places (44.1%, 95%CI, 43.5–44.7%). Within the current model, if more than 85% of people had adopted both behaviors, the initial *R*_*t*_ could have been less than 1.

**Conclusion:**

Our model results indicate that wearing face masks and avoiding crowded places could have potentially significant suppressive impacts on influenza.

## 1. Introduction

Many studies warned that repeated COVID-19 outbreaks are expected to happen and the number of infections and deaths could become even worse during winter (1–5). Besides the relaxation of social distancing during winter holidays, seasonal influenza, which commonly circulates during wintertime, may facilitate the transmission and mortality of COVID-19 if both severe acute respiratory syndrome coronavirus 2 (SARS-CoV-2) and influenza virus spread at the same time (6–9). In fact, many places have seen surges in COVID-19 infections in 2020/2021 winter (10–12). Since many cities have reopened after the vaccine has been distributed, it is important to know whether individual precautionary behaviors (such as avoiding crowded places and wearing face masks) without strict social distancing rules can prevent an influenza outbreak. How to prevent influenza outbreak or the co-circulation with COVID-19 are major tasks for World Health Organization (13, 14).

During the early spread of COVID-19 in China, many Hong Kong residents began to wear face masks mainly in public transport and avoid going to crowded places voluntarily. Due to the high influx of travelers from mainland China, Hong Kong faced and acknowledged an extremely high risk during the early spread. After WHO made an announcement of the initial spreading of COVID-19 in Wuhan on January 14, 2020 (15), people in Hong Kong perceived the risk of infection and changed their behavior immediately. Cowling et al. (16) showed that the number of people avoiding crowded places and wearing face masks increased between January and February 2020. Their first survey (January 20–23) was conducted immediately after the announcement by WHO about noting limited human-to-human transmission and the First-Level Public Health Emergency Response in China (15, 17). Their second survey (February 11–14) was conducted after the first local (community) transmission event was confirmed in Hong Kong on February 4. Few cases sporadically occurred up to early March, indicating that the first significant COVID-19 outbreak began. Because seasonal influenza incidence was progressively reducing during these survey periods soon after its initial rapid growth, it is likely that this unexpected reduction in the incidence of influenza was due to the behavioral changes in response to the potential COVID-19 spread.

These precautionary behavioral changes during January and February 2020 showed a more relaxed restriction than formal social distancing rules or other non-pharmaceutical interventions (NPIs) implemented later (e.g., the first group gathering ban was effective from March 29, 2020). By comparing the effective reproduction number (*R*_*t*_) in influenza along with these differences in the precautionary behaviors, the corresponding effects can be quantified, which provide important insights to understand whether an influenza outbreak can be controlled using less intensive social distancing restrictions without huge socioeconomic impacts. Furthermore, whether individuals practice precautionary measures or choose to be vaccinated largely depends on their risk perception, relating to a complex decision-making process (18, 19). Knowing the impacts of these behavioral changes help to forecast the possible epidemic situations after reopening.

Timely public health decision-making often needs to be made during an outbreak. However, the methods of estimating parameters, such as *R*_*t*_, of traditional susceptible-infectious-recovered (SIR) equations under Bayesian framework (e.g., Markov chain Monte Carlo (20), particle filtering (21, 22), etc.) are usually time-consuming. Alternatively, the time-series susceptible-infectious-recovered (TS-SIR) model provides a computationally inexpensive way to model the transmission dynamics as the parameters can be estimated through a generalized linear model (GLM) (23–25). Compared with frequentist approaches, Bayesian approaches to modeling and inference of infectious disease dynamics have the advantage that latent parameters (e.g., actual numbers of uninfected (susceptible) and infected individuals) and their uncertainties can be seamlessly accounted for (26). To further reduce the computational load from traditional methods for Bayesian inference, some approximation methods such as integrated nested Laplace approximations (INLA) approach can be applied (27).

The aim of our study was to identify the relationship between precautionary behaviors (e.g., wearing face masks and avoiding crowded places) and the reduction in influenza transmissibility. We adopted a TS-SIR model to estimate the *R*_*t*_ in influenza seasons by using a Bayesian approach with INLA. TS-SIR was transformed to a GLM with Poisson regression. After considering the effect of underreporting and separating the effect from herd immunity, we were able i) to quantify the effects of wearing face masks and avoiding crowded places throughout an outbreak and ii) to identify the required percentage of people adopting such precautionary behaviors that could suppress the outbreak.

## 2. Materials and methods

### 2.1. Data collection

**Figure 1:**
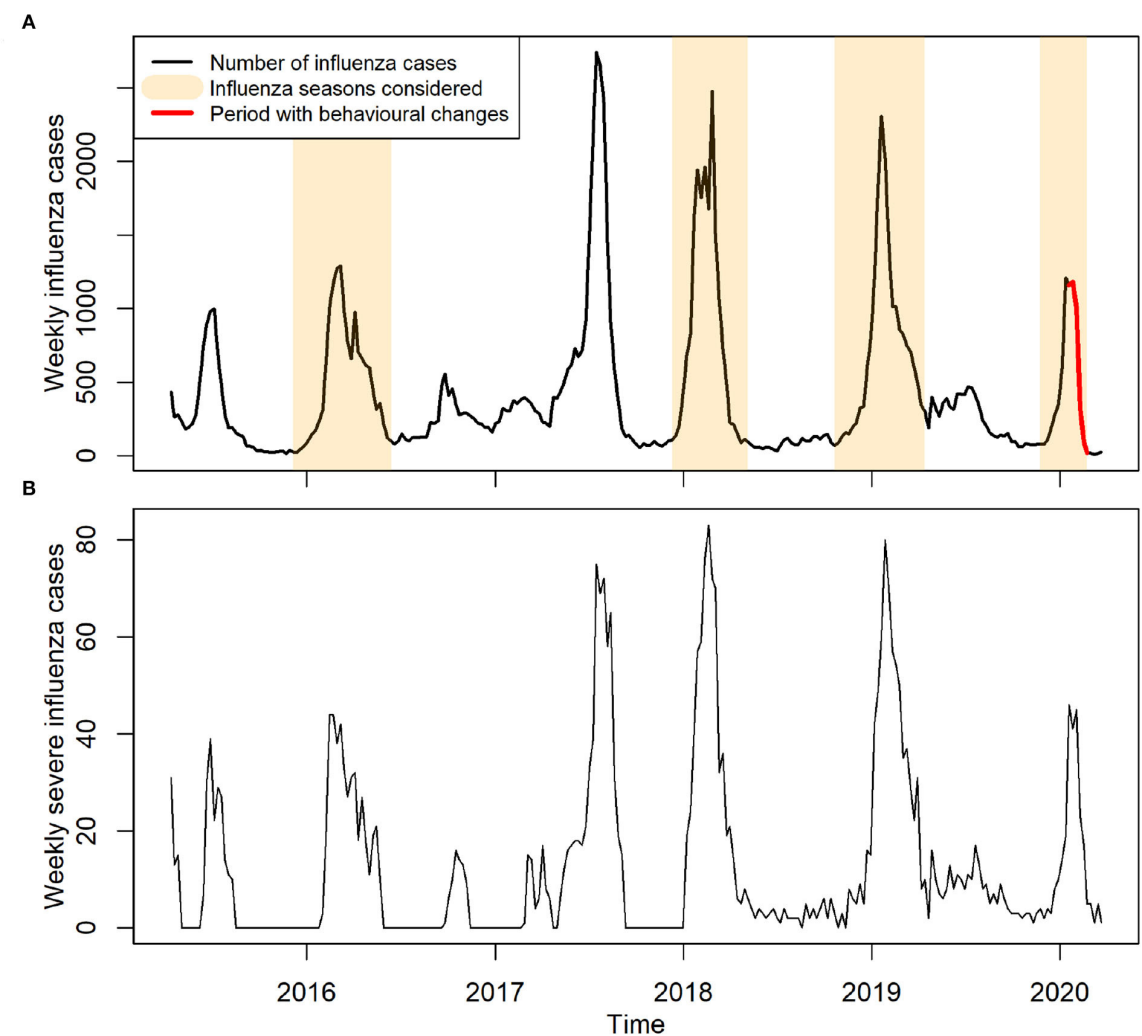
Reported cases of influenza in Hong Kong between years 2015 and early 2020. **(A)** Weekly reported influenza cases. Influenza seasons considered for model training are highlighted in light brown color, indicating an ordinary influenza period. Red color indicates the period with precautionary behavioral changes. **(B)** Weekly reported severe influenza cases.

The weekly reported influenza cases in Hong Kong from April 12, 2015 to March 22, 2020 were obtained from the Centre for Health Protection (CHP) (28). Only outbreaks during regular winter seasons were collected for our study (Figure 1A).

### 2.2. Modeling

On January 14, 2020, WHO made an announcement of COVID-19 outbreak, and shortly afterward, China declared a first-level public health emergency response (15, 17). Hence, within the study period of 2019/20 seasonal influenza outbreak (Figure 1A), we defined the period from the start of the outbreak (November 24, 2019) up to January 12, 2020 before the majority of Hong Kong residents knew the existence of COVID-19 as an ordinary influenza period. This ordinary period also included the winter influenza seasons during the years 2015–2016, 2017–2018, and 2018–2019. We included these previous seasons to increase the statistical power and obtain robust estimates of the baseline reporting rate. We did not consider the year 2016–2017 in our training set as there was no obvious winter seasonal peak.

Three phases with different transmission patterns were observed in the 2019–2020 winter influenza season (i.e., the growing, plateau, and decline periods). These phases were correlated to the stages of COVID-19 spread in Hong Kong. The **ordinary phase** (Phase 1) indicated the period before COVID-19. In addition to the ordinary phase (corresponding to the growing period of the outbreak), we further split the influenza season after January 12, 2020 into two other phases: the **awareness phase** (Phase 2), from January 12 to February 2, when people received the announcement given by WHO, corresponding to the plateau period; and the **spreading phase** (Phase 3), from February 2 to February 23, during which local community transmission occurred in Hong Kong, corresponding to the decline period.

***Time-Series Susceptible-Infectious-Recovered (TS-SIR) model***

Effective reproduction number *R*_*t*_ at time *t* was calculated as follows:


(1) $$R_{t}=R_{0}\times C_{j}\times (\frac{S_{t}}{N}),$$

where *R*_0_ is the basic reproduction number and *C*_*j*_ is the contact ratio, a ratio of the contact rate during phase *j* compared to the pre-pandemic period (Phase 1; *j* = 1). The baseline contact ratio for the pre-pandemic period was fixed at *C*_1_ = 1. *S*_*t*_ is the susceptible population at time *t* and *N* is the population in Hong Kong. Since official population data were only reported in 2016 and 2021 from the Census and Statistics Department of Hong Kong (29), we assumed the population growth to be linear between 2016 and 2021, that is, the population was 7,336,585 in 2016, 7,365,931 in 2018, 7,380,605 in 2019, and 7,395,278 in 2020. We defined the effects of behavioral changes on transmissibility in phase *j* (Φ_*j*_) as the percentage of reduction in contact ratio, in which the effect of herd immunity (i.e., the effect contributed by the reduction in susceptible population over time) is removed:


(2) $$\Phi_{j}=(1-C_{j})\times 100\%.$$

While many of the epidemiological models used for influenza modeling are conventional compartmental models (i.e., SIR model), an alternative, though related, model is the TS-SIR model (24), which transforms the conventional model to a GLM, a classic regression approach. In this study, we adopted a TS-SIR model with reference to Imai et al. (24) to capture the transmission dynamics for influenza and we considered different reporting rates at different periods of time due to public's risk perception amid COVID-19:


(3) $$\begin{array}{l} \frac{Y_{t}}{\rho_{j}}=R_{t-1}^{T_{c}}\times \frac{Y_{t-1}}{\rho_{j}}\\ \;=(R_{0}\times C_{j}\times \frac{S_{t-1}}{N})T_{c}\times \frac{Y_{t-1}}{\rho_{j}}\\ \;=(R_{0}\times C_{j}\times \frac{N-\sum_{i=0}^{t-1}Y_{i}/\rho_{j}}{N})T_{c}\times \frac{Y_{t-1}}{\rho_{j}}, \end{array}$$

where *Y*_*t*_ is the reported incidence at time *t* and *S*_*t*−1_ is the susceptible population at time *t*−1; we considered the susceptible population equal to total population minus the cumulative incidence within an influenza season, i.e., $S_{t-1}=N-\overset{t-1}{\underset{i=0}{\sum }}Y_{i}/\rho_{j}$, ρ_*j*_ is the reporting rate at Phase *j*. Because weekly influenza data are published by CHP in Hong Kong, the unit of *t* is week (and *t* = 1, 2, …). To calculate the number of infected cases generated from a single infected case after a unit of time, a time scale *T*_*c*_ relative to the generation time of 3.5 days is introduced (30), which is calculated as 7/3.5 = 2.

Equation (3) can be transformed to a GLM with Poisson distribution (see Supplementary material for details), such that *Y*_*t*_/ρ_*j*_~Poisson(μ_*t*_), where μ_*t*_ denotes the expected value of the weekly influenza cases. We had $(\log(\mu_{t})=\log(\frac{Y_{t-1}}{\rho_{j}})+T_{c}\log(R_{0}\times C_{j})-\frac{T_{c}\times \sum_{i=0}^{t-1}Y_{i}/\rho_{j}}{N}$.

To estimate parameters, we first obtained *R*_0_ and ρ_1_ during the ordinary period (which includes the winter influenza seasons during 2015–16, 2017–18, 2018–19, and 2019–20 up to January 12, 2020). Then, we obtained ρ_2_, *C*_2_, ρ_3_, and *C*_3_, subsequently, by modeling the situations in Phase 2 and Phase 3. The detailed procedures for model fitting can be found in Supplementary material.

We estimated the effects of wearing face masks and avoiding crowded places on the contact ratio defined in our model. The percentage of reduction in contact ratio was used to represent the percent reduction in *R*_*t*_ while excluding the impact from the herd immunity (see Equation 2). We assumed that these two effects are independent and additive; thus, we have


(4) $$\begin{array}{l} \Phi_{j}=\phi_{sd}\left(x_{j,sd}-x_{1,sd}\right)+\phi_{m}\left(x_{j,m}-x_{1,m}\right), \end{array}$$

where Φ_*j*_ is the overall percent reduction of contact ratio in Phase 2 and Phase 3 (*j* = 2, 3), which was previously estimated from Equations 1 and 2; *x*_*j, sd*_ and *x*_*j, m*_ are the percentages of population avoiding crowded places and wearing face masks in Phase *j*, respectively. *x*_1, *sd*_ and *x*_1, *m*_ are their baseline percentages (the estimates of the baseline percentages come from our survey, see Results Section for details). ϕ_*sd*_ and ϕ_*m*_ are parameters indicating the effectiveness of individual behaviors. The product of ϕ and *x* was referred as the effect on contact ratio in total population. To account for the uncertainty in the extent of avoiding crowded places and wearing face masks at different periods of time, we assumed that the number of survey participants avoiding crowded places or wearing face masks followed a binomial distribution (the number of trials is equal to the population in Hong Kong in 2020, with different probabilities in the different phases according to the mean percentages in the surveys. We adopted a bootstrap approach to capture the uncertainty in model parameters (see Supplementary material for details). The code for the abovementioned models can be found at https://github.com/hy39/ts-sir-flu.

## 3. Results

**Figure 2:**
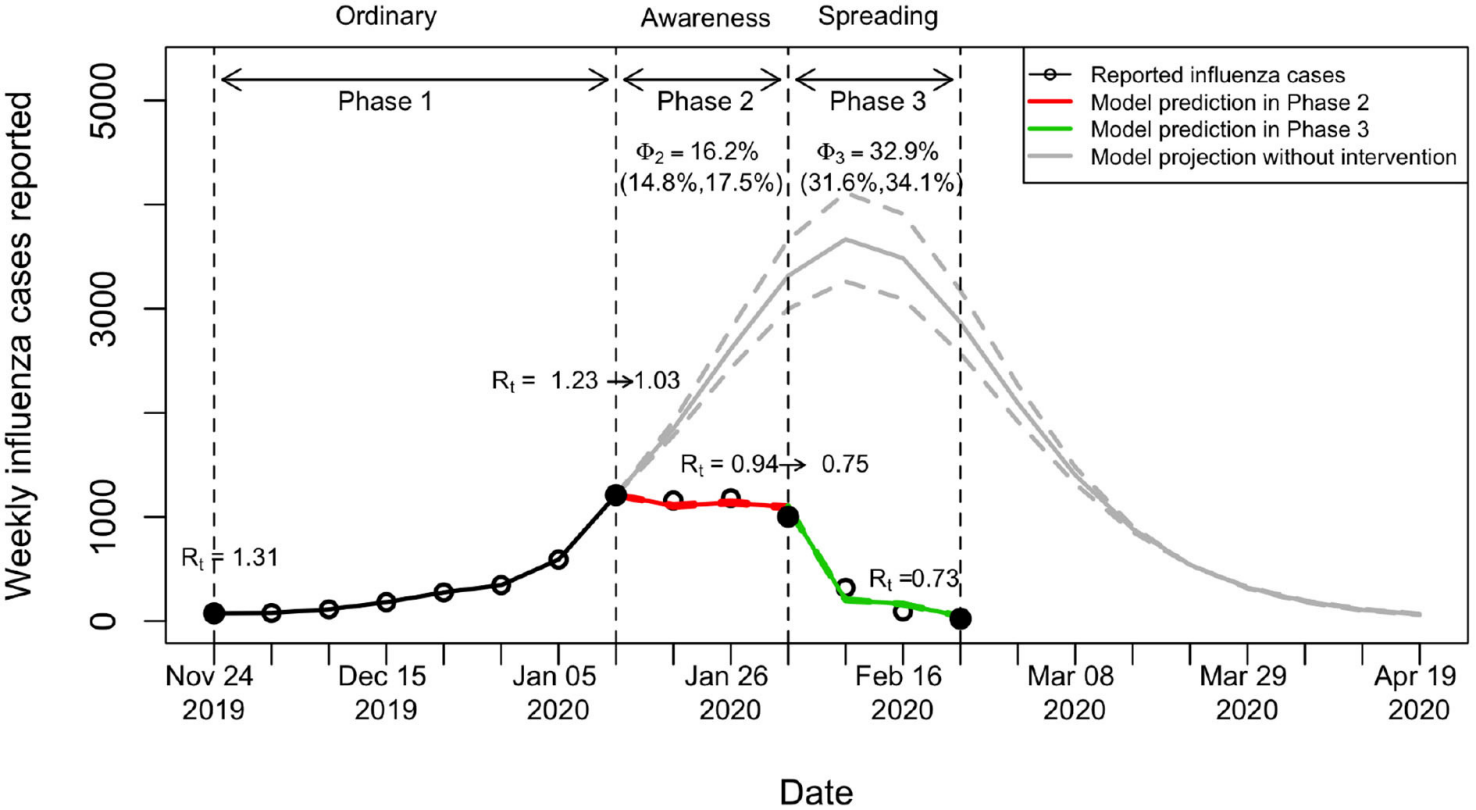
Model prediction of influenza cases in 2019–20 winter influenza season in Hong Kong. *R*_*t*_ at the boundaries (the second and third dashed lines) between phases were estimated from the model taking account of changes in both behavior and herd immunity. The prediction in Phase 2 (Awareness: the period immediately after people were aware of the existence of COVID-19) is shown in red. The prediction in Phase 3 (Spreading: the period immediately after local COVID-19 cases began to spread) is shown in green. The prediction intervals in Phase 2 and 3 are shown with narrow intervals. Φ denotes the percentage of reduction in *R*_*t*_ at Phase 2 and 3 compared to Phase 1, resulting from the changes in behavior only (i.e., excluding the effects from herd immunity).

**Figure 3:**
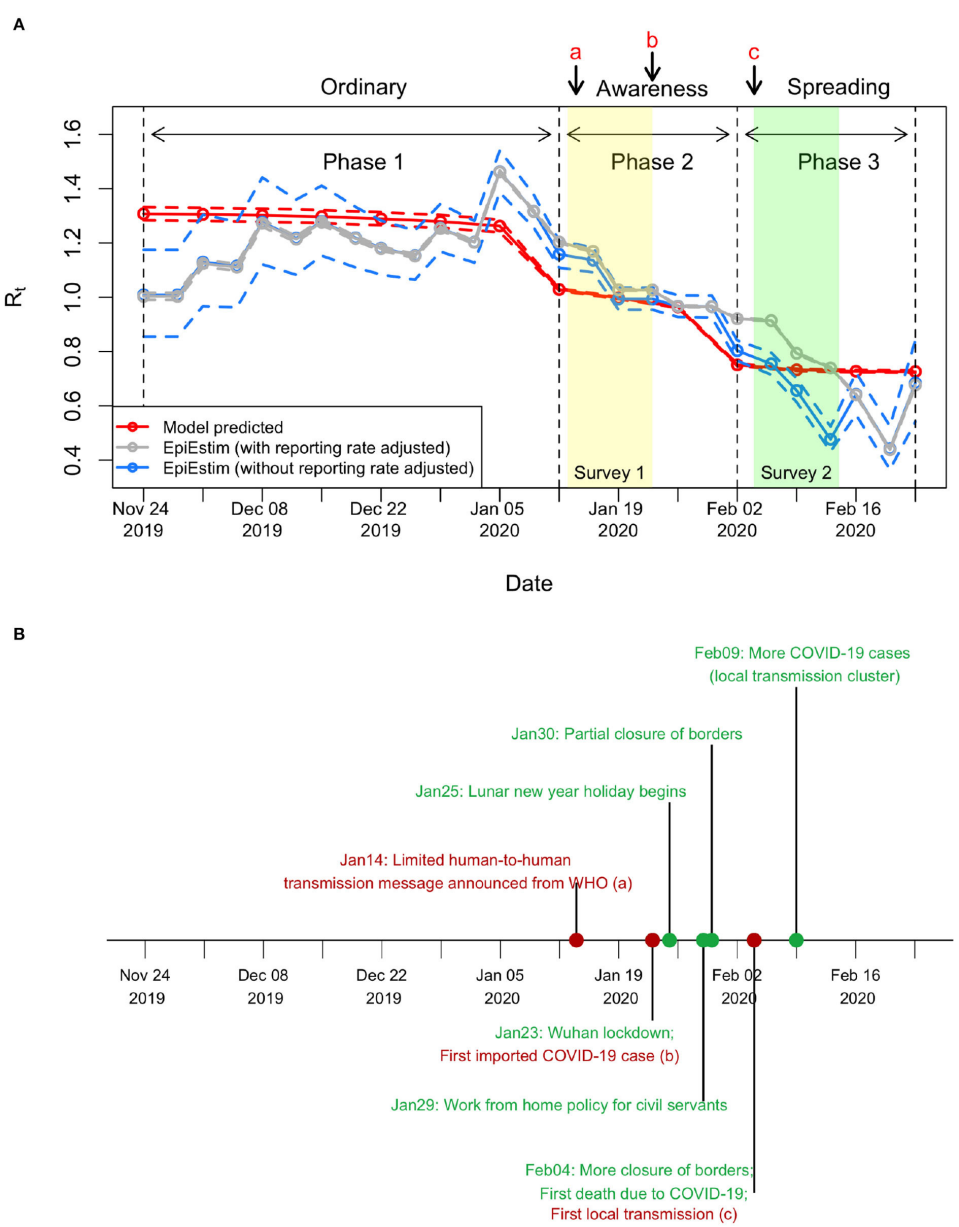
Changes in *R*_*t*_ before COVID-19 outbreak. **(A)** Estimated *R*_*t*_ in 2019/20 winter influenza season. The mean *R*_*t*_ was estimated as 1.29 (95%CI, 1.27–1.32) in Phase 1, reduced to 1.00 (95%CI, 0.99–1.00) in Phase 2, and further reduced to 0.73 (95%CI, 0.73–0.74) in Phase 3. In applying EpiEstim package, the number of influenza cases per week was converted into cases per 3.5 days by linear interpolation on the cumulative influenza cases, which equals to roughly one generation time of influenza (30). For other settings in applying the EpiEstim package, the serial interval was a gamma distribution with the mean equal to 3.5 days and the standard deviation equal to 1 day, and the window size was 2 weeks. **(B)** Timeline about COVID-19. Important events are shown in red, while other events are shown in green.

To quantify the effects of behavioral changes (in response to the initial spread of COVID-19) on influenza transmissibility, we classified the 2019–20 winter influenza season into three different phases based on the pattern of influenza activity as mentioned in the Section 2 (Figure 2): Phases 1, 2, and 3 show the growth, plateau, and decline phases of the dynamics, respectively. Comparing influenza activity in year 2019–2020 with the previous seasons, the growth became apparently limited after Phase 1 and then reduced significantly without showing a typical curvature of epidemic peak. Presumably, this unusual pattern was due to the human behavioral changes associated with people's risk perception on certain critical public health events (i.e., COVID-19 spreading) (Figure 3B). Hence, we correlated these three phases to different epidemic statuses of COVID-19, namely ordinary, awareness, and spreading phases (see Section 2 and Figure 2).

To estimate the effects of behavioral changes on the transmissibility, we adopted a TS-SIR model by taking account of the herd immunity changes. Our model captured the dynamics across the three phases well. The number of influenza cases stopped growing after people avoided crowded places and wore face masks. Compared with the projection of cases without the effects of behavioral changes (i.e., under ordinary transmission dynamics), the number of cases began to decline at least 4 weeks earlier and the total number of reported cases until February 23 was reduced by 78.8% (Figure 2).

Initial *R*_*t*_, also called the basic reproduction number *R*_0_, was estimated to be 1.37 (95%CI, 1.35–1.4). In 2020 winter influenza season, the *R*_*t*_ reduced slightly from 1.31 to 1.23 during Phase 1 (Figures 2, 3A), which was mainly caused by the increase in herd immunity after the infected cases were recovered. After the risk of COVID-19 transmission has been noticed, the *R*_*t*_ on January 12 reduced from 1.23 (end of Phase 1) to 1.03 (start of Phase 2), with an effect of behavioral changes Φ_2_ (i.e., the percentage of reduction in *R*_*t*_ after excluding the effects of herd immunity; see Section 2) being 16.2% (95%CI, 14.8–17.5%) in this phase. The *R*_*t*_ on February 2 further reduced from 0.94 (end of Phase 2) to 0.75 (start of Phase 3), with an overall effect of behavioral changes Φ_3_ being 32.9% (95%CI, 31.6–34.1%) in this phase. In Phase 3, the *R*_*t*_ slightly reduced to 0.73 at the end. The prediction intervals in Phase 2 and Phase 3 are narrow because the uncertainty of adjusted reporting rates at the corresponding phases was small (see Supplementary material and subsequent paragraphs for details). The results showed that the reductions in transmissibility were primarily due to the behavioral changes against COVID-19 and only partially due to the increase in herd immunity (Figure 3A).

**Figure 4:**
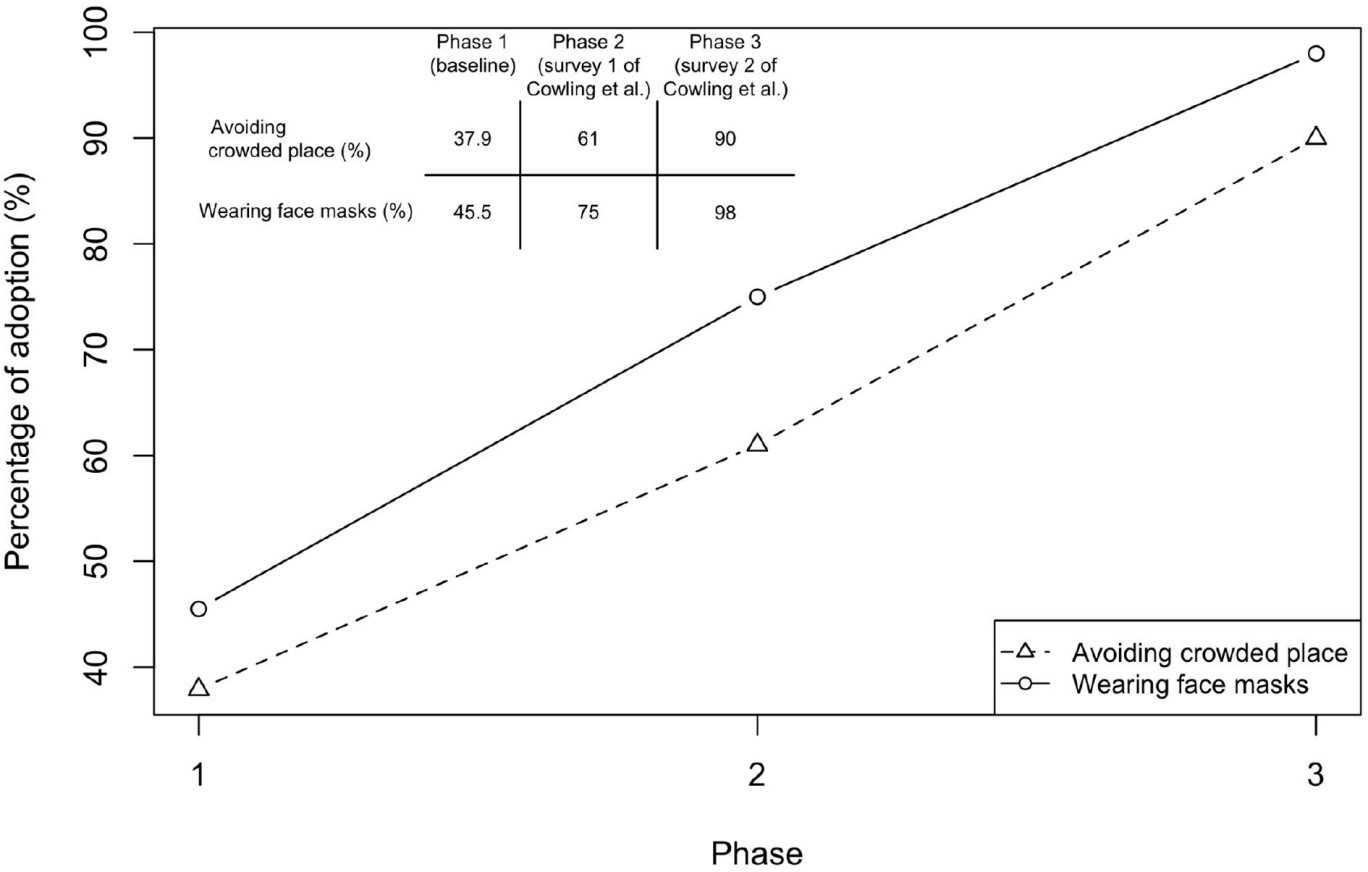
Changes in behavior in response to COVID-19. Results of Phase 1 were taken from a survey conducted during January 5–February 15, 2020 for assessing the baseline behavior taken to prevent influenza infection. Sample size is 66, with 45 females. Respondents were aged between 19 and 64. Three respondents have taken flu vaccination. Note that the survey questions only ask the possible measures in order to prevent influenza but not COVID-19. Hence, the results indicate the baseline behavior before COVID-19 emerged (Avoiding crowded places: 37.9%, 95%CI, 26.2–49.6%; Wearing face masks: 45.5%, 95%CI, 33.5–57.5%). Results of Phases 2 (Cowling Survey 1) and 3 (Cowling Survey 2) were taken from a previous study conducted by Cowling et al. (16)) before NPIs were implemented in Hong Kong. Cowling Survey 1 (January 20-23) was conducted immediately after the WHO made an announcement on January 14, 2020 and when China declared a first-level public health emergency response (January 20, 2020) (15, 17) (sample size is 1008; Avoiding crowded places: 61%, 95%CI, 57.2–65.4%; Wearing face masks: 75%, 95%CI, 70.4–78.6%). Cowling Survey 2 (February 11-14) was conducted after the first community transmission event was confirmed in Hong Kong (sample size is 1000; Avoiding crowded places: 90%, 95%CI, 86.2–94.2%; Wearing face masks: 98%, 95%CI, 93.5–100%).

We next quantified the effects of different behaviors. The survey during the baseline showed that 37.9% of people would avoid crowded places and 45.5% would wear face masks for preventing influenza infection (Figure 4). The behavioral changes in the subsequent phases were revealed by the study of Cowling et al. (16) (Figure 4), in which two surveys were conducted immediately after Phase 2 and 3 began (Figure 3A). The percentage of people avoiding crowded places increased from 60% to 90%, while the percentage for wearing face masks increased from 75 to 98%. At that time, social distancing rules, such as gathering ban, have not been implemented by the Hong Kong government yet. Both avoiding crowded places and wearing face masks were precautionary behaviors triggered by individual's risk perception.

We estimated the effects of these two behavioral changes after separating the effect of herd immunity (see Section 2). The results showed that wearing face masks was associated with an 17.4% reduction in *R*_*t*_ (the coefficient is 0.174, 95%CI, 0.163–0.183) (Table 1). The effect of avoiding crowded places was 44.1% (the coefficient is 0.441, 95%CI, 0.435 to 0.447). When nobody wears face masks or avoids crowded places, the initial *R*_*t*_ was 1.71 (95%CI, 1.707–1.715), which was higher than our previous estimate of 1.37. This is because a fraction of people have adopted preventive measures for influenza (Figure 4). To reduce *R*_0_ to below one, more than 84.3% (95%CI, 84.1–84.5%) of people have to wear face masks and avoid crowded places.

**Table 1:** Impacts of precautionary behaviors in influenza control and transmissibility from our model.

**Impact**	**Value (95%CI)**
(%) Effectiveness of avoiding crowded places	44.1 (43.5, 44.7)
(%) Effectiveness of wearing face masks	17.4 (16.3, 18.3)
Estimated *R*_0_ when nobody	
wears face masks or avoids crowded places	1.71 (1.707, 1.715)
Percentage of people required to adopt precautionary	
behaviors in order to lower *R*_0_ to below 1	84.3% (84.1%, 84.5%)

Note that we addressed the concerns of underreporting due to COVID-19 by adjusting the reporting rates in different phases (see Supplementary material for details) with the ratio of the severe influenza cases to total reported influenza cases (Table 2 and Figure 1B). The ordinary reporting rate was estimated as 0.0065 (95%CI, 0.0064–0.0067), and the adjusted reporting rate dropped to 0.0057 (95%CI, 0.0056–0.0059) in Phase 2 and further to 0.0022 (95%CI, 0.0021–0.0022) in Phase 3. There was a reduction in reporting rate (0.0008 and 0.0043, respectively, for Phase 2 and Phase 3, compared with Phase 1) across the three phases, which conforms to the expectation that fewer patients with influenza visit hospitals or clinics under the risk of COVID-19.

**Table 2:** Ratios of severe cases to the total reported cases in several winter influenza seasons.

**Year**	**Ratio (%)**
2016	3.1
2018	3.5
2019	3.8
2020 - Phase 2	4.0
2020 - Phase 3	10.0

The ratios were applied to adjust the reporting rates in Phase 2 and Phase 3 of 2019/2020 winter influenza season.

Our results were compared with the *R*_*t*_ estimated using data on the number of observed new cases with a statistical method based on renewal function (EpiEstim package) (31) (Figure 3A). The comparison showed that the *R*_*t*_ estimations from both methods were consistent. However, the *R*_*t*_ from the EpiEstim package showed larger variations within each phase than our predictions. Before the spread of COVID-19 (i.e., Phase 1), the *R*_*t*_ from EpiEstim are similar with and without reporting rate adjusted (gray line and blue line, respectively, in Figure 3A). However, without adjusting reporting rate, the *R*_*t*_ from EpiEstim was lower than the *R*_*t*_ estimates with the reporting rate adjusted.

## 4. Discussion

The importance of wearing face masks on stopping COVID-19 spread through droplet or aerosol transmission has been addressed by the WHO (32). Although the effects of wearing face masks on preventing common respiratory virus infection, such as influenza or SARS-CoV-2, have been intensely discussed using empirical evidence from laboratory (33–36) or simulation studies (37), the evidence from the population study is little (38). We quantified the effects of wearing face masks and avoiding crowded places on seasonal influenza transmissibility during early COVID-19 spread period when human behavior changed. The results demonstrated that precautionary behavioral changes may have had a large impact on influenza transmission, even before strict social distancing rules were implemented. This gives important recommendation on the prevention of future influenza using NPIs.

Possibly because of the risk perception related to previous experience of SARS epidemic in 2003, the adoption rate of face masks in Hong Kong was high even before COVID-19 began to spread in the community [see Figure 4 from our data and the data revealed by the surveys from Cowling et al. (16) and Kwok et al. (39)]. Even though these spontaneous behavioral changes were less restricted than formal social distancing rules, our estimation, taking account of the underreporting of influenza cases, showed that *R*_*t*_ reduced from 1.31 to 0.73 (Figure 2). High risk perception may also affect the decision-making in vaccination. The complex relationships between behavioral changes and transmission dynamics can be modeled through the evolutionary game theory (18, 19), which is important in predicting and preparing for future outbreaks after reopening.

A reduction in the incidence of influenza has also been reported in mainland China during the early COVID-19 spread (40), which further supports the finding that the interventions implemented against COVID-19 significantly reduced influenza incidence. The interventions appeared to have different degrees of impact on influenza incidence than in Hong Kong, which was likely because of the start time of the influenza season. In mainland China, the influenza season began in November 2019 (40) but the COVID-19 interventions (first-level responses) were implemented in late January 2020, when the epidemic peak has already been reached. However, Hong Kong had an influenza season at a later time and the COVID-19-induced interventions or precautionary behaviors were adopted before the peak (Figure 2). Hence, the incidence of influenza incidence was less in Hong Kong.

Based on the data from Hong Kong, our results showed that wearing face masks could reduce seasonal influenza transmission by as much as 17.4% in the population, which is nearly half of the effect of avoiding crowded places (44.1%). Within our model, if more than 85% of the people had avoided crowded places and wore face masks, *R*_*t*_ could have reduced to below one. This evidence suggests that, without strong policy restrictions in social distancing (i.e., four-person gathering ban in public places or even a lockdown), the incidence of influenza can still be greatly reduced by simple behavioral changes. This highlights the need of future research on whether mandatory mask wearing policy in certain public places only (e.g., public transport or other crowded places) can significantly reduce influenza infection.

A TS-SIR model provides a convenient way to estimate epidemiological parameters using the classical GLM approach without losing the nonlinear effects in the conventional SIR model. To estimate the reproduction number accurately, our model took account of the change in reporting rate due to the outbreaks of COVID-19, with reference to the fact that people were reluctant to go to the clinic (81% (16) and 76% (39) of the respondents). Using the TS-SIR model, we were able to separate the changes in *R*_*t*_ due to both behavioral changes and the increase in herd immunity. This allowed us to quantify the changes in *R*_*t*_ caused by the behavioral changes using a classical statistical approach.

Nevertheless, there are some limitations in our study. In the proposed model, we assumed the population was random mixing without considering the effect of heterogeneous mixing (24). We mainly assumed that avoiding crowded places and wearing face masks were the major behaviors that could reduce the number of transmissions, because these transmissions mainly occur through the droplets released when an infected person sneezes, coughs, or speaks. In addition, in the effectiveness-behavior analysis (Equation 4), we checked whether confounding factors occurred. We found that the probability of wearing face masks is not conditional on avoiding crowded places, enabling us to use a simple additive approach to assess the individual effects. A larger sample indeed can increase the statistical power of the effectiveness-behavior analysis (i.e., the credible intervals of the resulted effects). However, due to time constraints, the sample size in our survey on Phase 1 behavior was limited. Furthermore, the surveyed behavioral changes were simply interpreted in the percentage of the population. Future studies should be conducted to quantify the duration of wearing face masks.

Preparing for the co-circulation of influenza and COVID-19 is critically important (14). While many countries lifted the requirements of social distancing and wearing face masks as COVID-19 vaccination rolled out and the omicron (B.1.1.529) outbreaks passed, these relaxations likely led to the rise of seasonal influenza infections. However, COVID-19 continues to spread with more than 1 million new cases per day globally (March 2022) (41). Some biotechnology companies have been developing a COVID-19-Influenza combination vaccine to provide protections from both illnesses at the same time (42, 43). To reduce the influenza incidence from a non-pharmaceutical perspective, we recommend that the idea of wearing face masks in certain public places and/or simple social distancing (i.e., avoiding crowded places) should be promoted. The effectiveness of such precautionary behaviors on seasonal influenza based on our study can also potentially give us hints for the recommendations of behavioral shift in dealing with future pandemics (44).

## Data availability statement

The original contributions presented in the study are included in the article/Supplementary material, further inquiries can be directed to the corresponding author. The code for the abovementioned models can be found at https://github.com/hy39/ts-sir-flu.

## Author contributions

H-YY and C-PL contributed to conception and design of the study and wrote the first draft of the manuscript. C-PL performed the analysis. ID, K-LT, MX, and M-HL wrote sections of the manuscript. All authors contributed to manuscript revision, read, and approved the submitted version.

## Funding

This research was funded by the Health and Medical Research Fund (COVID190215), the City University of Hong Kong (7200573 and 9610416), the Wellcome Trust and the Royal Society (213494/Z/18/Z), and Hong Kong Innovation and Technology Commission (InnoHK Project CIMDA).

## Conflict of interest

The authors declare that the research was conducted in the absence of any commercial or financial relationships that could be construed as a potential conflict of interest.

## Publisher's note

All claims expressed in this article are solely those of the authors and do not necessarily represent those of their affiliated organizations, or those of the publisher, the editors and the reviewers. Any product that may be evaluated in this article, or claim that may be made by its manufacturer, is not guaranteed or endorsed by the publisher.
