# Supplementary material for: Impact of early phase COVID-19 precautionary behaviors on seasonal influenza in Hong Kong: A time-series modeling approach
Source: Front Public Health. 2022 Nov 14;10:992697. doi: 10.3389/fpubh.2022.992697 (PMC9728392; doi:10.3389/fpubh.2022.992697)
Supplement: Supplementary file 1 [file Data_Sheet_1.pdf]

Supplementary Material

A1 TS-SIR MODEL FITTING

From Eq. 3, by taking logarithm of both sides and applying Maclaurin series approximation (proof is given in Section A2), it becomes:

$$\log(\frac{Y_t}{\rho_j}) = \log(\frac{Y_{t-1}}{\rho_j}) + T_c \log(R_0 \times C_j) - \frac{T_c \times \sum_{i=0}^{t-1} Y_i/\rho_j}{N}. \quad \text{(S1)}$$

In order to model each influenza outbreak within the ordinary period ($j = 1$, i.e., winter influenza seasons in 2015/16, 2017/18, 2018/19, and 2019/20 up to January 12, 2020), we included an indicator function to represent the transmissibility of each seasonal outbreak and assumed that the contact rates and reporting rates remained constant within this ordinary period, but became different from the period after January 12, 2020. Thus, the relative contact ratio C_1 is 1 as the baseline, and then Eq. S1 is reduced to Eq. S2:

$$\log(Y_{t,m}) = \log(Y_{t-1,m}) + T_c \log(R_0 + \sum_{m=1}^{3} R_m^* z(m)) - \frac{T_c \times (\eta Y_{0,m}^* + \sum_{i=0}^{t-1} Y_{i,m})/\rho_1}{N}, \quad \text{(S2)}$$

where $z(m)$ is the indicator function:

$$z(m) = \begin{cases} 1, & \text{if time } t \text{ belongs to the } m\text{-th season} \\ 0, & \text{otherwise} \end{cases}$$

and $m = 1, 2, 3$ referring to winter influenza seasons in 2015/16, 2017/18, 2018/19, respectively. $Y_{t,m}$ is the weekly reported incidence at time t within the m-th season. R_0 is the basic reproduction number in 2019/20 and R_m^* measures the difference between R_0 and that in particular influenza season. Therefore, the sum of R_0 and R_m^* denotes the basic reproduction number in the m-th season. To incorporate the effect of pre-existing immunity in each season before the outbreak starts, we assumed this immunity was only caused by infections during summer before each winter season. The effect of partial immunity produced from the preceding summer season was accounted for through R_m^*, e.g., larger immunity resulted from transmission in the preceding summer season results in a lower R_m^* in winter. $Y_{0,m}^*$ is the sum of summer incidence (between the end of the previous winter influenza season and the start of the current m-th season, excluding $Y_{0,m}$), denoting the pre-existing immunity before the outbreak grows. $Y_{0,m}$ is the weekly incidence one week before the first week of the m-th season. η is the efficacy of the immunity from preceding summer season, which is assumed to be 30%. The assumption is made with reference to the influenza vaccine efficacy (European Centre for Disease Prevention and Control, n.d.).

In fitting the above models (Eq. S2 and Eq. S1), we adopted a GLM with Poisson distribution under Bayesian framework. Let μ_t denotes the expected value of the weekly influenza cases with reporting rate

considered, then, we had $Y_t/\rho_j \sim \text{Poisson}(\mu_t)$ and $\log(\mu_t) = \log(\frac{Y_{t-1}}{\rho_j}) + T_c \log(R_0 \times C_j) - \frac{T_c \times \sum_{i=0}^{t-1} Y_i/\rho_j}{N}$. The Bayesian inference was performed using INLA (Rue et al., 2009) along with the R-INLA package (Rue et al., 2017).

We first estimated R_0 and ρ_1 during the ordinary period using Eq. S2. In such fitting, we fixed the coefficient of $\log(Y_{t-1})$ to 1. After, we adjusted the reporting rate (estimating ρ_2 and ρ_3) through the number of severe influenza cases (details are given in Section A4). Then we estimated the relative contact ratios (C_2 and C_3) during the time following the ordinary period (i.e. Phase 2 and Phase 3, respectively) using Eq. S1. In such fitting, we fixed the coefficient of $\log(\frac{Y_{t-1}}{\rho_j})$ to 1 and adopted a Gaussian prior on the coefficient of $-\frac{T_c \times \sum_{i=0}^{t-1} Y_i/\rho_j}{N}$ to account for the uncertainties in reporting rate.

A2 Proof of Maclaurin series approximation

To obtain Eq. S1 from Eq. 3, we applied Maclaurin series approximation to the first approximation and got:

$$\log(\frac{N - \sum_{i=0}^{t-1} Y_i/\rho_j}{N}) = \log(1 - \frac{\sum_{i=0}^{t-1} Y_i/\rho_j}{N}) \approx -\frac{\sum_{i=0}^{t-1} Y_i/\rho_j}{N}. \tag{S3}$$

Proof:

For $|x| < 1$,

$$\begin{aligned} f(x) &= \log(1-x) \\ &\approx f(0) + \frac{f'(0)}{!1}(x-0) \\ &= \log(1) + (-1)x \\ &= -x, \end{aligned} \tag{S4}$$

Substituting $x = \frac{\sum_{i=0}^{t-1} Y_i/\rho_j}{N}$ and $\left|\frac{\sum_{i=0}^{t-1} Y_i/\rho_j}{N}\right| < 1$,

$$\log(1 - \frac{\sum_{i=0}^{t-1} Y_i/\rho_j}{N}) \approx -\frac{\sum_{i=0}^{t-1} Y_i/\rho_j}{N}. \tag{S5}$$

A3 Bootstrapping in fitting effectiveness-behaviour model

For each bootstrap sample, we considered two phases, Phase 2 and Phase 3 (i.e., two equations from Eq. 4). We sampled one observation from the corresponding distributions of the behaviours at Phase 1, Phase 2, and Phase 3, respectively, as the predictor variables, and we sampled one observation from

the corresponding distributions of Φ at Phase 2 and Phase 3, respectively, as the response variable. And we solved the equations to obtain the model parameters. We repeated this process for 10000 times to obtain a distribution of the effects of avoiding crowded places and wearing face masks, respectively. We considered ranked samples with orderly matching (i.e., at each phase, we generated 10000 samples from each distribution, arranged them in ascending order, and paired the samples according to their ranking), so that the sampling variation systematically occurred between each phase.

A4 Adjusting reporting rate

To address the concern on the issue of under-reporting (reduced number of clinic or hospital visits) during COVID-19 outbreak, we used the situation of severe influenza cases as a proxy to adjust the reporting rate. The underlying assumption is that the under-reporting issue that happened during COVID-19 outbreak would not occur in severe influenza cases as the patients were very sick and could not avoid going for medical care. In this sense, the ratio of the severe cases to the total reported cases can be used to adjust the reporting rate in each phase. The number of severe influenza cases in Hong Kong was collected from the CHP data (Centre for Health Protection, Department of Health, Hong Kong SAR, 2020) and is shown in Figure 1B.

A multiplicative correction factor (CF) was proposed to adjust the reporting rate in 2020 after COVID-19. The CF_j at Phase j is defined as follows:

$$CF_j = \frac{1}{r_j/r_1}, \tag{S6}$$

where r_j is the ratio of severe to total reported cases within a time period belonging to Phase j when reporting rate is adjusted, r_1 is the ordinary ratio of severe to total reported cases, which equals the mean ratio in 2015/16, 2017/18 and 2018/19 winter influenza seasons (illustrated in Figure 1A).

The adjusted reporting rate is obtained by:

$$\rho_j = \rho_1 \times CF_j, \tag{S7}$$

where ρ_1 is the ordinary reporting rate estimated using the ordinary winter seasons in 2015/16, 2017/18 and 2018/19 (illustrated in Figure 1A).

REFERENCES

European Centre for Disease Prevention and Control. Influenza vaccine effectiveness. *European Centre for Disease Prevention and Control* (n.d.).

Rue H, Martino S, Chopin N. Approximate Bayesian inference for latent Gaussian models using integrated nested Laplace approximations (with discussion). *Journal of the Royal Statistical Society B* **71** (2009) 319–392.

Rue H, Riebler AI, Sørbye SH, Illian JB, Simpson DP, Lindgren FK. Bayesian computing with INLA: A review. *Annual Reviews of Statistics and Its Applications* **4** (2017) 395–421.

Centre for Health Protection, Department of Health, Hong Kong SAR. Flu express (2020).
